# Essential Updates 2023/2024: Minimally Invasive Surgery for Biliary Tract Cancer

**DOI:** 10.1002/ags3.70073

**Published:** 2025-08-03

**Authors:** Osamu Itano, Takuya Minagawa

**Affiliations:** ^1^ Department of Hepato‐Biliary‐Pancreatic and Gastrointestinal Surgery, School of Medicine International University of Health and Welfare Chiba Japan

**Keywords:** bile duct cancer, gallbladder cancer, laparoscopic liver resection, minimally invasive liver resection, robot‐assisted liver resection

## Abstract

Minimally invasive surgery (MIS) for biliary tract cancer (BTC) has gained traction with advancements in laparoscopic and robotic techniques. However, its oncological impact remains uncertain. This review highlights key studies from 2023 and 2024 on MIS for BTC, including distal bile duct, hilar bile duct, gallbladder, and intrahepatic bile duct cancer. Although feasibility and safety are supported in experienced centers, long‐term prognostic equivalence to open surgery remains debatable. Further studies are required to clarify surgical and oncological outcomes.

## 1 Introduction

Biliary tract cancer (BTC) encompasses intrahepatic, perihilar, and distal cholangiocarcinoma, along with gallbladder carcinoma. Despite regional variations, its incidence is rising [1, 2]. Surgical resection remains the cornerstone of curative treatment. Compared with other gastrointestinal cancers, minimally invasive surgery (MIS) adoption for BTC has been slower because of technical and oncological challenges. However, the proliferation of robotic surgical systems is expanding its application. This review highlights key 2023 and 2024 reports on MIS for BTC.

## 2 Distal Cholangiocarcinoma (dCCA)

dCCA occurs between the cystic duct junction and ampulla of Vater [3, 4], comprising 20%–40% of cholangiocarcinomas [5]. Pancreaticoduodenectomy (PD) is the standard treatment. Laparoscopic PD (LPD) has gained global use since the 2000s [6]. Advancements in operative techniques and perioperative care have improved postoperative survival [7]. MIS for dCCA may facilitate faster recovery and improve chemotherapy adherence [8, 9]. However, concerns remain regarding lymph node retrieval and pancreatic fistula. Particularly, MIS may yield fewer tumor‐positive lymph nodes [10], and inadequate nodal assessment can impact prognosis [11]. Robotic PD (RPD) is being explored to address these limitations.

In 2023 and 2024, four key studies [12, 13, 14, 15] analyzed MIS for dCCA. Two were retrospective comparative studies using propensity score matching (PSM), and two were systematic reviews. Uijterwijk et al. [12] compared 97 minimally invasive PD (MIPD) cases (37 RPD, 60 LPD) with 381 open PD (OPD) cases. MIPD was associated with less blood loss (300 vs. 420 mL; *p* = 0.025), longer operative time (453 vs. 336 min; *p* < 0.001), and fewer surgical site infections (8% vs. 19%; *p* = 0.042). Overall survival (OS) and disease‐free survival (DFS) were comparable. RPD also yielded comparable lymph node retrieval (14 vs. 14; *p* = 0.214) and major complication rates (23% vs. 20%; *p* = 0.811). Zhu et al. [13] compared 45 LPD cases with 60 OPD cases. LPD was associated with lower blood loss (302 vs. 505 mL; *p* = 0.03) and superior OS (68 vs. 47 months; *p* = 0.002). These findings are summarized in Table 1.

**TABLE 1:** Summary of recent MIS studies in distal cholangiocarcinoma.

Articles	Type of article	Patient number	Operation time (min)	Estimated blood loss (mL)	Complication rate	R0 resection (%)	Retrieved lymph nodes	Overall survival	Disease‐free survival
Uijterwijk [12]	Single‐center, PSM	97 MIS (37 RS, 60 LS) vs. 381 OS	453 vs. 336 (median); *p* < 0.001	300 vs. 420 (median); *p* = 0.025	23% vs. 20% (C‐D ≥ IIIb); *p* = 0.811	NA	14 vs. 14 (median); *p* = 0.214	35% vs. 24% (5 years); *p* = 0.224	25% vs. 21% (5 years); *p* = 0.344
Zhu [13]	Single‐center, PSM	45 LS vs. 60 OS	NA	302 vs. 505 (mean); *p* = 0.03	NA	93 vs. 93; *p* = 1.0	14 vs. 10 (mean); *p* = 0.076	68 vs. 47 months (median); *p* = 0.002	NA

Abbreviations: C‐D, Clavien‐Dindo classification; DFS, disease‐free survival; LS, laparoscopic surgery; MIS, minimally invasive surgery; NA, not applicable; OS, open surgery; PSM, propensity score matching; RS, robotic surgery.

Uijterwijk et al. [14] conducted a meta‐analysis of 1949 patients, including 529 dCCA cases. Although MIPD had longer operative times, other short‐term outcomes were comparable. However, DFS for dCCA was significantly lower in MIPD (hazard ratio [HR] = 1.7; *p* = 0.045), warranting further investigation. Domene et al. [15] analyzed 1349 patients (405 MIPD, 944 OPD) and found no significant differences in transfusions, complications, or mortality. These studies suggest that MIPD is feasible for dCCA in experienced centers, offering potential recovery and morbidity benefits. However, concerns about lymph node retrieval and long‐term oncological outcomes remain. Further prospective trials are needed to establish its role in BTC treatment.

## 3 Perihilar Cholangiocarcinoma (pCCA)

pCCA is an aggressive malignancy with poor prognoses. Surgical resection remains the only curative option [16, 17]. R0 resection significantly improves survival, with 5‐year survival rates reaching 30%–67% [18, 19, 20]. Surgery typically involves hepatectomy with en‐bloc extrahepatic bile duct resection and regional lymphadenectomy, often requiring vascular resection and reconstruction. Even in experienced centers, the 90‐day mortality after a major hepatectomy for pCCA can reach 10% [21, 22, 23].

MIS for pCCA has been explored since the early 2000s. Laparoscopic surgery is feasible with comparable short‐term outcomes to open surgery [24, 25]. However, it is technically demanding and limited to highly selected patients. Robotic surgery offers enhanced dexterity and precision [26], but evidence remains limited, with only nine studies reported in a 2022 systematic review.

In 2023 and 2024, 12 studies on MIS for pCCA [27, 28, 29, 30, 31, 32, 33, 34, 35, 36, 37, 38] were published. All laparoscopic reports originated from China, mostly single‐center experiences with fewer than 50 cases. Liu et al. [27] introduced a novel “left‐liver‐first anterior radical modular orthotopic right hemihepatectomy” (Lap‐Larmorh), reporting a statistically shorter operation time. Yin et al. [28] found no significant differences in surgical or oncologic outcomes between laparoscopic and open surgery. Similarly, Wang et al. [29] found no significant differences in intraoperative blood loss and operative time between both approaches. Du et al. [30] reported a 2.2% rate of Clavien‐Dindo grade IIIa complications, no grade IIIb or higher, and DFS rates of 74%, 61%, and 40% at 1, 2, and 3 years, respectively.

Two Chinese multicenter studies analyzed laparoscopic surgery for pCCA. Wang et al. [31] used propensity score matching across 11 centers, finding similar short‐term outcomes to open surgery and shorter hospital stays (15 vs. 18 days, *p* = 0.0007). Qin et al. [32] analyzed data from 10 centers, showing comparable morbidity and OS, with laparoscopic surgery associated with fewer severe complications and shorter stays.

Interest in robotic surgery for pCCA has grown, with six reports published in 2023 and 2024. Magistri et al. [33] reported 93% R0 resections, no 90‐day mortality, and > 50% 3‐year survival. D'Hondt et al. [34] reported no 90‐day mortality and a 338‐min median operative time. Liu et al. [35] compared robotic and laparoscopic resections, finding similar perioperative outcomes, with robotic surgery offering shorter, higher‐quality anastomoses. Huang et al. [36] compared robotic and open resections, reporting longer operative time for robotics (548 vs. 353 min, *p* = 0.004) but more lymph nodes retrieved (11 vs. 5, *p* = 0.010). These results are summarized in Table 2.

**TABLE 2:** Summary of recent MIS studies in perihilar cholangiocarcinoma.

Articles	Type of article	Patient number	Operation time (min)	Estimated blood loss (mL)	Complication rate	R0 resection (%)	Retrieved lymph nodes	Overall survival	Disease‐free survival
Liu et al. [27]	Single‐center	14 LS	373 (median)	500 (median)	71% (not defined)	93	NA	40% (3 years)	NA
Yin et al. [28]	Single‐center	40 LS vs. 28 OS	469 vs. 440 (mean); *p* = 0.154	350 vs. 550 (mean); *p* = 0.062	70% vs. 75% (C‐D ≥ I); *p* = 0.786	80 vs. 79; *p* = 0.554	NA	38% vs. 20% (3 years); *p* = 0.358	43% vs. 42% (3 years); *p* = 0.945
Wang et al. [29]	Single‐center	16 LS vs. 9 OS	601 vs. 472 (mean); *p* = 0.121	559 vs. 189 (mean); *p* = 0.115	13% vs. 11% (C‐D ≥ III); NA	88 vs. 56; *p* = 0.142	8 vs. 5 (mean); *p* = 0.028	NA	NA
Du et al. [30]	Single‐center (case series)	44 LS	285 (mean)	360 (mean)	2% (C‐D ≥ III)	89	8 (median)	30 months^a^ (median)	40%^a^ (3 years)
Wang et al. [31]	Multicenter, PSM	256 LS vs. 389 OS	359 vs. 342 (mean); *p* = 0.151	300 vs. 300 (median); *p* = 0.573	12% vs. 23% (C‐D ≥ III); *p* = 0.006	82 vs. 84; *p* = 0.526	12 vs. 11 (mean); *p* = 0.542	NA	NA
Qin et al. [32]	Multicenter, PSM	161 LS vs. 306 OS	360 vs. 356 (median); *p* = 0.914	300 vs. 300 (median); *p* = 0.775	12% vs. 19% (C‐D ≥ III); *p* = 0.2	94 vs. 88; *p* = 0.176	8 vs. 8 (median); *p* = 0.57	NA vs. 35 months (median); *p* = 0.915	NA
Magistri et al. [33]	Single‐center (case series)	14 RS	490 (median)	150 (median)	21% (C‐D ≥ III)	93	19 (median)	> 50% (3 years)	NA
D'Hondt et al. [34]	Single‐center (case series)	10 RS	338 (median)	110 (median)	NA	90	9 (median)	NA	NA
Liu et al. [35]	Single‐center, PSM	10 RS vs. 36 LS	385 vs. 374 (mean); *p* = 0.931	200 vs. 310 (median); *p* = 0.109	20% vs. 30% (C‐D ≥ III); *p* = 0.682	NA	NA	NA	NA
Huang et al. [36]	Single‐center, PSM	10 RS vs. 76 OS	548 vs. 353 (median); *p* = 0.004	125 vs. 350 (median); *p* = 0.067	20% vs. 50% (C‐D ≥ III); *p* = 0.235	90 vs. 80; *p* = 0.64	11 vs. 5 (median); *p* = 0.010	NA	NA

Abbreviations: C‐D, Clavien‐Dindo classification; DFS, disease‐free survival; LS, laparoscopic surgery; NA, not applicable; OS, open surgery; PSM, propensity score matching; RS, robotic surgery.

^a^ For R0‐resected cases.

Berardi et al. [37] conducted a systematic review of 18 studies (372 MIS cases: 310 laparoscopic, 62 robotic), concluding that MIS for pCCA is feasible and safe. Munir et al. [38] analyzed national cancer database records, defining “textbook oncologic outcome” as R0 resection with adequate lymphadenectomy, no prolonged hospitalization, no 90‐day mortality, and appropriate adjuvant therapy. They found MIS achieved better lymphadenectomy (robotic: 71.4%, laparoscopic: 41.3%, open: 40.8%, *p* = 0.019), with no significant OS difference.

Although publication bias should be considered, current evidence suggests that MIS for pCCA is feasible and safe when performed by experienced surgeons. However, achieving adequate lymph node dissection (LND) and R0 resection remains a major challenge, and further studies are needed to determine whether these oncologic standards can be consistently met in minimally invasive settings. Careful patient selection is essential when considering MIS for BTC. In particular, MIS may not be suitable for advanced pCCA requiring vascular resection or extensive biliary reconstruction, for which open surgery remains the standard. Current evidence supports the use of MIS primarily in highly selected cases at experienced centers.

## 4 Gallbladder Cancer (GBC)

GBC is the most common biliary tract malignancy with a poor prognosis, often diagnosed at advanced stages [39, 40, 41]. Surgical resection remains the only curative option for localized GBC. MIS has expanded, demonstrating feasibility and short‐term benefits, though concerns remain about its safety, feasibility, and oncologic outcomes [42, 43, 44]. The standard surgical approach varies by T stage, ranging from simple cholecystectomy for Tis‐T1a to extended cholecystectomy with liver resection and lymphadenectomy for T1b or greater [45]. Given the difficulty of accurate preoperative T staging, Itano et al. [43] proposed a laparoscopic approach combined with intraoperative pathological examinations to improve diagnostic accuracy and tailor the surgical procedure.

Eighteen studies on MIS for GBC [46, 47, 48, 49, 50, 51, 52, 53, 54, 55, 56, 57, 58, 59, 60, 61, 62, 63] were published in 2023 and 2024, evaluating surgical and oncologic outcomes. Several single‐center analyses compared laparoscopic radical resection with open surgery, showing that laparoscopic surgery results in reduced blood loss, shorter hospital stays, and comparable survival outcomes. Fontana et al. [46] compared 18 laparoscopic versus 57 open liver segment 4b5 resections, reporting significantly lower blood loss (100 vs. 237 mL, *p* = 0.001), shorter hospital stays (4 vs. 8 days, *p* < 0.001), and higher lymph node harvests (9 vs. 7, *p* = 0.026), with no differences in resection margins, morbidity, bile leakage, and survival. Umemura et al. [47] reported laparoscopic extended cholecystectomy (L‐EC) with en‐bloc lymphadenectomy as feasible for clinical T2 GBC. Cheng et al. [48] confirmed the safety and feasibility of laparoscopic lymphadenectomy, with low complication rates and favorable prognosis. Jin et al. [49] found comparable OS and DFS between laparoscopic and open extended re‐resections for incidental GBC. Wu et al. [50] performed a PSM analysis, showing that laparoscopic surgery reduced operation time, blood loss, drain time, and hospital stay without increasing complications, achieving a prognosis similar to open surgery.

Minagawa et al. [51] conducted a multi‐institutional retrospective study of laparoscopic radical GBC resection in 129 patients (82 confirmed GBC), reporting a 269‐min median operation time, 30‐mL median blood loss, and 79% and 87% 5‐year OS and DFS rates, respectively.

Three meta‐analyses evaluated laparoscopic versus open surgery. Karjol et al. [52] found higher OS in the open group despite no differences in recurrence, operative time, blood loss, lymph node yield, and complications. Ahmed et al. [53] concluded that laparoscopic surgery is non‐inferior oncologically but had higher port‐site recurrences and yielded fewer lymph nodes. Li et al. [54] reported better 2‐year survival, lower blood loss, shorter hospitalization, and fewer complications in laparoscopic cases but noted variations in oncologic outcomes attributable to differences in included studies.

To enhance intraoperative decision‐making, Banh et al. [55] evaluated frozen section analysis in 37 suspected GBC cases, finding 100% sensitivity and specificity in detecting malignancy, which enables tailored surgical approaches.

Six studies (two meta‐analyses, two multicenter studies, and two single‐center reports) analyzed robotic surgery for GBC. Cho et al. [56] and Tschuor et al. [57] reported that robotic resection resulted in lower blood loss and shorter hospital stays than open surgery, with similar oncologic outcomes. Sohn et al. [58] conducted a South Korean multicenter study comparing minimally invasive extended cholecystectomy (MI‐EC) and open EC (O‐EC), with subgroup analyses of L‐EC and robotic (R‐EC) approaches. MI‐EC had longer operative time but shorter hospital stays. L‐EC took longer than R‐EC; however, perioperative and survival outcomes were comparable, though R‐EC remained costly. Ielpo et al. [59] performed an international multicenter study with 575 patients, comparing robotic, laparoscopic, and open approaches. After PSM, the robotic group had higher median lymph node harvests (7 vs. 5 open, 7 vs. 4 laparoscopic, *p* < 0.015), shorter Pringle maneuver time (38 vs. 59 min laparoscopic, *p* = 0.0034), lower conversion rates (3% vs. 14% laparoscopic, *p* = 0.005), and less blood loss. OS and DFS were similar across groups. These results are summarized in Table 3.

**TABLE 3:** Summary of recent MIS studies in gallbladder cancer.

Articles	Type of article	Patient number	Operation time (min)	Estimated blood loss (mL)	Complication rate	R0 resection (%)	Retrieved lymph nodes	Overall survival	Disease‐free survival
Fontana et al. [46]	Single‐center	18 LS vs. 57 OS	355 vs. 259 (median); *p* < 0.001	100 vs. 237 (median); *p* = 0.001	6% vs. 11% (C‐D ≥ III); *p* = 0.532	94 vs. 93; *p* = 0.829	9 vs. 7 (median); *p* = 0.026	94% vs. 65% (3 years); *p* = 0.57	89% vs. 65% (3 years); *p* = 0.46
Umemura et al. [47]	Single‐center (case series)	5 LS	151 (mean)	46 (mean)	20% (C‐D ≥ III)	100	9 (mean)	NA	NA
Cheng et al. [48]	Single‐center (case series)	39 LS	251 (median)	197 (median)	NA (not described in detail)	NA	14 (T1b, mean), 13 (T2, mean), 9 (T3, mean)	100% (T1b, 3 years), 74% (T2, 3 years), 38% (T3, 3 years)	100% (T1b, 2 years), 80% (T2, 2 years), 25% (T3, 2 years)
Jin et al. [49]	Single‐center	84 LS vs. 117 OS	253 vs. 198 (median); *p* < 0.001	310 vs. 530 (median); *p* < 0.001	8% vs. 14% (C‐D ≥ III); *p* = 0.98	61 vs. 57; *p* = 0.4	8 vs. 8 (median); *p* = 0.68	74 vs. 77 months (median); *p* = 0.67	73 vs. 71 months (median); *p* = 0.18
Wu et al. [50]	Single‐center, PSM	44 LS vs. 119 OS	128 vs. 195 (mean); *p* < 0.001	50 vs. 100 (median); *p* = 0.002	3% vs. 3% (C‐D ≥ III)	94 vs. 97; *p* = 1	NA	69% vs. 68% (3 years); *p* = 0.65	69% vs. 66% (3 years); *p* = 0.663
Minagawa et al. [51]	Multicenter	129 LS	269 (median)	30 (median)	2% (C‐D ≥ III)	96	2 (D1, median), 7 (D2, median)	79% (5 years)	87% (5 years)
Cho et al. [56]	Single‐center, PSM	41 RS vs. 84 OS	202 vs. 195 (mean); *p* = 0.48	383 vs. 717 (mean); *p* = 0.02	12% vs. 10% (C‐D ≥ III); *p* > 0.99	100 vs. 100; *p* > 0.99	7 vs. 8 (mean); *p* = 0.338	92% vs. 97% (5 years); *p* = 0.807	73% vs. 78% (5 years); *p* = 0.991
Tschuor et al. [57]	Single‐center	20 RS vs. 23 OS	193 vs. 208 (median); *p* = 0.604	150 vs. 350 (median); *p* = 0.002	10% vs. 17% (C‐D ≥ III); *p* = 0.485	80 vs. 83; *p* = 0.826	5 vs. 3 (median); *p* = 0.189	61% vs. 61% (2 years); *p* = 0.438	NA
Sohn et al. [58]	Multicenter, PSM	69 MIS (29 RS, 40 LS) vs. 308 OS	240 vs. 190 (mean); *p* < 0.001	513 vs. 389 (mean); *p* = 0.142	9% vs. 12% (C‐D ≥ III); *p* = 0.57	92 vs. 93; *p* = 0.891	7 vs. 9 (mean); *p* = 0.052	57% vs. 69% (5 years); *p* = 0.227	59% vs. 63% (5 years); *p* = 0.475
Ielpo et al. [59]	Multicenter, PSM	123 RS vs. 98 OS	260 vs. 275 (mean); *p* = 0.27	183 vs. 320 (mean); *p* = 0.002	7% vs. 12% (C‐D ≥ III); *p* = 0.227	89 vs. 84; *p* = 0.469	7 vs. 5 (median); *p* = 0.015	49 vs. 46 months (median)	32 vs. 32 months (median)
Ielpo et al. [59]	Multicenter, PSM	123 RS vs. 155 LS	256 vs. 257 (mean); *p* = 0.95	177 vs. 205 (mean); *p* = 0.001	7% vs. 8% (C‐D ≥ III); *p* = 0.788	88 vs. 81; *p* = 0.3	7 vs. 4 (median); *p* < 0.001	49 vs. 43 months (median)	32 vs. 31 months (median)

Abbreviations: C‐D, Clavien‐Dindo classification; DFS, disease‐free survival; LS, laparoscopic surgery; MIS, minimally invasive surgery; NA, not applicable; OS, open surgery; PSM, propensity score matching; RS, robotic surgery.

Chee et al. [60] conducted a systematic review and network meta‐analysis including 5883 patients from 32 studies, finding that MIS led to shorter hospitalization, less blood loss, and fewer complications than open surgery, with comparable oncologic outcomes. However, no clear advantage was found between robotic and laparoscopic surgery. Mellado et al. [61] reviewed comparative analyses of robotic and open surgery in 353 patients from five studies, concluding that robotic surgery had shorter operative times, hospital stays, and blood loss with similar oncologic outcomes. Although robotic surgery shows advantages in surgical outcomes, larger studies are needed to determine oncologic benefits.

## 5 Intrahepatic Cholangiocarcinoma (ICC)

ICC accounts for approximately 10%–20% of liver malignancies and generally presents as a large tumor with a poor prognosis [64]. The 5‐year OS for ICC is 20%–40%, significantly lower than the 50%–70% observed in hepatocellular carcinoma (HCC). ICC also shows higher rates of lymph node involvement (28% vs. 8%) and larger tumors (≥ 5 cm: 64% vs. 46%) than HCC [65]. The only curative treatment is radical resection with negative margins (R0) [66, 67]; moreover, owing to the high rate of lymph node involvement (approximately 40%), routine LND retrieving at least six lymph nodes is recommended for adequate staging [68, 69].

Although laparoscopic liver resection (LLR) has shown benefits in HCC and colorectal liver metastases [70, 71, 72], its role in ICC remains limited owing to the complexity of major hepatectomy and LND. Despite this, PSM studies and meta‐analyses suggest LLR is feasible and safe in high‐volume centers [73, 74]. However, achieving effective LND remains challenging. Some studies report lower LND rates with LLR than open liver resection (OLR) [75], whereas others suggest that laparoscopic magnification may help identify lymph nodes [76]. Although controversial, hilar LND remains essential for staging and guiding adjuvant therapy and may improve OS in node‐negative patients [77, 78].

Between 2023 and 2024, eight studies [79, 80, 81, 82, 83, 84, 85, 86] reported on MIS for ICC: three retrospective PSM analyses, two systematic reviews and meta‐analyses, and one prospective trial evaluating robotic surgery versus open surgery. Kim et al. [79] analyzed 49 LLR and 170 OLR cases via PSM, showing shorter hospital stays (8 vs. 12 days, *p* = 0.002) and lower ICU admission rates (12% vs. 33%, *p* = 0.038) in the LLR group, with no significant differences in OS or recurrence‐free survival (RFS). Sahakyan et al. [80] reported lower complication (30% vs. 52%, *p* = 0.025) and reoperation rates (4% vs. 16%, *p* = 0.046) in LLR despite a lower LND rate (20% vs. 60%, *p* < 0.01). Shen et al. [81] found LLR associated with reduced intraoperative blood transfusion (30% vs. 69%, *p* < 0.001) and blood loss (200 vs. 500 mL, *p* = 0.003), whereas OS and DFS remained comparable to OLR.

Two systematic reviews and meta‐analyses evaluated laparoscopic versus open surgery. Aliseda et al. [82] (six studies, 1042 patients) reported that LLR was associated with reduced bleeding (−161 mL), transfusion (odd ratios [OR] = 0.41), major complications (OR = 0.60), and hospital stay (−3.2 days). Hu et al. [83] (10 trials) reinforced these findings. Although LND and major hepatectomy rates were lower in LLR, R0 resection rates were higher (relative risk [RR] = 1.05), with fewer major complications (RR = 0.72). OS (HR = 0.91), DFS (HR = 0.95), and RFS (HR = 0.80) were comparable between LLR and OLR.

Shapera et al. [84] reported non‐inferiority of robotic liver resection versus OLR regarding oncological outcomes, with significantly lower estimated blood loss and no significant difference in tumor margin distance or OS. Although the accuracy of LND in LLR is an issue that needs to be improved, the short‐term outcomes of MIS for ICC are generally favorable. These results are summarized in Table 4.

**TABLE 4:** Summary of recent MIS studies in intrahepatic cholangiocarcinoma.

Articles	Type of article	Patient number	Operation time (min)	Estimated blood loss (mL)	Complication rate	R0 resection (%)	Retrieved lymph nodes	Overall survival	Disease‐free survival
Kim et al. [79]	Single‐center, PSM	49 LS vs. 170 OS	NA	260 vs. 385 (mean); *p* = 0.12	7% vs. 7% (C‐D ≥ III); *p* = 1	98 vs. 98; *p* = 1	NA	40% vs. 48% (5 years); *p* = 0.133	51% vs. 45% (5 years); *p* = 0.453
Sahakyan et al. [80]	Multicenter, PSM	50 LS vs. 86 OS	209 vs. 294 (mean); *p* < 0.01	NA	24% vs. 38% (C‐D ≥ III); *p* = 0.13	84 vs. 84; *p* = 0.93	1 vs. 2 (median); *p* = 0.002	47% vs. 43% (5 years); *p* = 0.43	40% vs. 38% (3 years); *p* = 0.9
Shen et al. [81]	Single‐center, PSM	127 LS vs. 43 OS	238 vs. 238 (median); *p* = 0.687	200 vs. 500 (median); *p* = 0.003	9% vs. 26% (C‐D ≥ III); *p* = 0.017	93 vs. 91; *p* = 0.795	NA	41% vs. 34% (5 years); *p* = 0.061	33% vs. 32% (5 years); *p* = 0.31
Shapera et al. [84]	Single‐center	15 RS vs. 19 OS	331 vs. 356 (mean); *p* = 0.7	100 vs. 420 (mean); *p* = 0.024	13% vs. 26% (C‐D ≥ III); *p* = 0.36	87 vs. 63; *p* = 0.129	6 vs. 6 (mean); *p* = 1.0	22 vs. 15 months (median); *p* = 0.243	NA

Abbreviations: C‐D, Clavien‐Dindo classification; DFS, disease‐free survival; LS, laparoscopic surgery; NA, not applicable; PSM, propensity score matching; RS, robotic surgery; OS, open surgery.

Given the importance of proper lymph node staging in ICC, as emphasized in recent guidelines, the lower rate of LND in MIS remains a significant concern. Future research should focus on optimizing techniques to ensure adequate nodal harvest and oncologic clearance.

## 6 Limitation

Although the growing body of literature provides valuable insight into the feasibility and safety of MIS for BTC, it is important to note that many of the studies in this review are retrospective, originate from single‐center experiences—particularly in China—and are conducted in high‐volume centers with significant MIS expertise. These factors may introduce selection bias, as well as regional and institutional variability, thereby limiting the generalizability of the findings to less experienced centers or broader patient populations.

## 7 Conclusion

We reviewed current topics on MIS for BTC. Robotic surgery may enhance surgical and oncological outcomes. However, high‐quality studies remain limited, and further research is necessary to establish robust evidence for clinical guidelines.

## Author Contributions

**Osamu Itano:** conceptualization, investigation, writing – original draft, writing – review and editing, methodology, supervision, data curation. **Takuya Minagawa:** conceptualization, investigation, writing – original draft, writing – review and editing, methodology, supervision, data curation.

## Ethics Statement

The authors have nothing to report.

## Conflicts of Interest

The authors declare no conflicts of interest. IO is an editorial board member of the *Annals of Gastroenterological Surgery*.

## Data Availability

The datasets used in this study are available from the corresponding author upon reasonable request.
